# Circulating plasma microRNA profiling in patients with polymyositis/dermatomyositis before and after treatment: miRNA may be associated with polymyositis/dermatomyositis

**DOI:** 10.1186/s41232-017-0058-1

**Published:** 2018-01-08

**Authors:** Takuya Hirai, Keigo Ikeda, Hiroshi Tsushima, Maki Fujishiro, Kunihiro Hayakawa, Yuko Yoshida, Shinji Morimoto, Ken Yamaji, Yoshinari Takasaki, Kenji Takamori, Naoto Tamura, Iwao Sekigawa

**Affiliations:** 1 Institute for Environmental and Gender-Specific Medicine, Juntendo University Graduate School of Medicine, Chiba, Japan; 2 Department of Internal Medicine and Rheumatology, Juntendo University Urayasu Hospital, 2-1-1 Tomioka Urayasu-shi, Chiba, 279-0021 Japan; 3 Department of Internal Medicine and Rheumatology, Juntendo University School of Medicine, Tokyo, Japan

**Keywords:** MicroRNA, Polymyositis, Dermatomyositis, Plasma

## Abstract

**Background:**

MicroRNAs (miRNAs) are involved in the regulation of key biological processes and have been implicated in various diseases, including autoimmune disorders. The pathogenesis of polymyositis (PM) and dermatomyositis (DM) is considered to be mediated by autoimmune reactions. To determine miRNA role in the development and progression of PM and DM, we performed plasma miRNA profiling in PM/DM patients before and after treatment.

**Methods:**

Total RNA was isolated from plasma of 10 patients before and after treatment with prednisolone, or, in case of prednisolone resistance or complications, with the combination of calcineurin inhibitors (cyclosporine or tacrolims) and/or pulse intravenous cyclophosphamide. The expression of miRNAs was determined using miRNA microarray and validated by qRT-PCR.

**Results:**

More differentially expressed miRNAs were found in plasma of DM patients compared to PM patients before and after treatment, and their profiles were different. Among the differentially expressed plasma miRNA identified by microarray, the levels of hsa-miR-4442 were confirmed by qRT-PCR to be significantly decreased by treatment. In addition, plasma hsa-miR-4442 content in active PM/DM significantly exceeded that in other active autoimmune diseases such as rheumatoid arthritis and systemic lupus erythematosus, as well as in healthy individuals. The level of plasma hsa-miR-4442 was positively correlated with Skeletal Disease Activity in MITAX (Myositis Intention to Treat Activity Index).

**Conclusion:**

This is the first report describing plasma miRNA expression profiles in PM/DM patients. The present data suggest that plasma levels of miRNAs may be associated with polymyositis/dermatomyositis and hsa-miR-4442 could be used as a biomarker for PM/DM diagnosis and/or disease activity.

**Electronic supplementary material:**

The online version of this article (10.1186/s41232-017-0058-1) contains supplementary material, which is available to authorized users.

## Background

Polymyositis (PM) is an idiopathic inflammatory disease characterized by the degeneration of the muscles, while dermatomyositis (DM) is polymyositis accompanied by skin inflammation manifested by heliotrope eyelids and Gottron’s signs which are red papules erupting on the finger joints. The pathogenesis of PM/DM is considered to be mediated by autoimmune response triggered by various causes, including cancer, and the disease can overlap with other autoimmune disorders such as systemic lupus erythematosus (SLE), rheumatoid arthritis (RA), and systemic sclerosis. However, the exact cause of PM/DM is still unclear.

Micro (mi)RNAs are small noncoding RNAs about 22 nucleotides in length that usually function as negative regulators of target mRNA translation by binding to mRNA 3′-untranslated region (UTR) [1]. miRNAs are involved in the regulation of key biological processes, including immune response and cell differentiation, proliferation, and apoptosis. The pathogenic role of miRNAs has been intensely studied in malignant diseases as well as in autoimmune disorders [2, 3]. miRNAs have been found in plasma and other body fluids of humans, indicating their high stability in the extracellular environment and suggesting that they may control cell-to-cell communication in health and disease. Highly stable extracellular miRNAs circulating in blood can be delivered by various cargo molecules such as high-density lipoproteins, nucleophosmin, and Argonaute 2 protein, to recipient cells where they regulate the expression of key proteins involved in disease pathogenesis [4–7]. Therefore, extracellular miRNAs present in blood circulation have attracted attention as disease biomarkers [8]. Furthermore, miRNA profiling of body fluids, including plasma and serum, has revealed significant differences in the spectrum and concentration of miRNAs between blood and tissues [9], suggesting that blood miRNA content may reflect specific disease-associated processes.

There are some reports about the changes of miRNA level in skin, muscle, or serum of PM/DM patients [10], but miRNA concentration in plasma has not been investigated. Based on recent reports, we also expected that more abundant microRNAs will be collected from exosomes and lipoproteins contained in plasma [11]. In addition, our previous high throughput studies of patients with autoimmune conditions before and after treatment revealed that serum/plasma contained biomarkers of disease pathogenesis and progression [12–14], suggesting that plasma may also be a source of PM/DM markers, including miRNA.

To test this hypothesis, here, we examined the changes in plasma miRNA profiles of patients with PM/DM before and after treatment.

## Methods

### Patients and samples

Plasma samples were obtained from four patients with PM and four patients with DM before and after treatment (active and inactive phase, respectively) to use in microarray. In order to validate the expression by qRT-PCR, we added samples from one PM patient and one DM patient. Clinicopathological characteristics of the patients enrolled in this study are shown in Table 1. All the patients fulfilled the criteria of Bohan and Peter, which include symmetrical muscle weakness, myositis, increase in serum skeletal muscle enzymes, characteristic electromyogram, and typical rash [15, 16]. The patients were analyzed for malignant tumors by computerized tomography and gastrointestinal endoscopy, and they did not have malignant tumors. All the patients received therapy with prednisolone were treated by the combination of calcineurin inhibitors (cyclosporine or tacrolims) and/or intravenous cyclophosphamide pulse therapy. Post treatment samples were obtained from patients who improved muscle symptoms. We investigated the Myositis Intention to Treat Activity Index (MITAX), CK, ALD, LDH, ESR as disease activity evaluation. MITAX is modified from the BILAG approach to assess disease activity in lupus [17]. MITAX assesses specific manifestations in seven organ systems (constitutional, cutaneous, skeletal, gastrointestinal, pulmonary, cardiac, and muscle systems). For MITAX assessment, three to nine criteria related to symptoms, physical findings, or laboratory abnormalities in each of the seven organ systems are assessed and converted into a score that range from zero to nine. The total disease activity score is calculated by summing the score of each organ system. Healthy individuals (seven women and three men, age 24–48 years old), RA patients (10 women, 51–82 years old), and SLE patients (11 women and 1 man, 17–54 years old) served as controls. RA patients were treated with conventional synthetic disease-modifying anti-rheumatic drugs (csDMARDs) or biologics in addition to csDMARDs, and the mean of DAS-ESR was 5.26. They were scheduled to introduce or to switch the biologics because they were ineffective. SLE patients were the onset or the recurrence, and the mean of SLEDAI scores was 17.4.

**Table 1:** Clinicopathological characteristics of PM/DM patients

				Clinical manifestations		Muscle strength (MMT)		Muscle enzymes			
	Patients	Age (years)	Sex	Cutaneous involvement	Pulmonary involvement	Upper limbs	Lower limbs	CK (IU/L)	ALD (IU/L)	LDH (IU/L)	Therapy
PM	P01	52	F	–	IP	4	4	1038	20.8	472	PSL 30 mg/day CyA 150 mg/day
	P02	83	F	–	–	4	4	8359	90.4	1224	PSL 40 mg/day TAC 2 mg/day
	P03	45	F	–	IP	4	4	417	21.2	404	PSL 40 mg/day TAC 3 mg/day IVCY 500 mg/4 weeks
	P04	17	F	–	–	4	4	2737	37.4	448	PSL 50 mg/day TAC 3 mg/day
	P05	46	M	–	–	5	5	2451	17.6	497	PSL 40 mg/day TAC 3 mg/day IVCY 500 mg/4 weeks
DM	D01	32	F	H, G, E	IP	4	3	6213	147	569	PSL 50 mg/day CyA 150 mg/day
	D02	18	F	E	–	4	4	17,662	59.1	1349	PSL 50 mg/day TAC 3 mg/day
	D03	62	M	H, G, E	–	4	4	7026	54	955	PSL 80 mg/day TAC 3 mg/day
	D04	40	F	G, E	IP	4	4	4246	106.7	703	PSL 50 mg/day TAC 3 mg/day
	D05	65	M	H, G, E	–	5	5	1176	18.5	410	PSL 60 mg/day IVCY 500 mg/4 weeks

*PM* polymyositis, *DM* dermatomyositis, *H* heliotrope rash, *G* Gottron’s sign, *E* erythema of elbows and/or knees, *IP* interstitial pneumonia, *MMT* manual muscle test, *CK* creatine kinase, *ALD* aldolase, *LDH* lactic dehydrogenase, *PSL* prednisolone, *CyA* cyclosporine, *TAC* tacrolims, *IVCY* intravenous cyclosphosphamide

### miRNA array analysis

Total RNA was extracted from 300 μl of plasma obtained from four PM and four DM patients using the 3D-Gene™ RNA extraction kit (Toray Industries Inc., Tokyo, Japan) and miRNeasy Mini Kit (Qiagen, Venlo, The Netherlands). The extracted RNA was labeled using the 3D-Gene miRNA labeling kit (Toray Industries Inc.), and the labeled targets were then hybridized to a 3D-Gene Human miRNA 4-plex chip (V21_V1.0.0, Toray Industries Inc.). Hybridization images were scanned in a GenePix4400A device (Molecular Devices, Sunnyvale, CA, USA), and miRNA expression was assessed based on signal intensity calculated as the median of foreground signals minus the mean of negative control signals + 2SD. As there was no definite internal control for plasma miRNA, the obtained data were normalized to the median intensity levels of miR-762, miR-3665, miR-3960, and miR-4516 using a per-chip 95th percentile method.

### Quantitative reverse transcription polymerase chain reaction

Total RNA was extracted from 200 μl of individual plasma samples using ISOGEN II (Nippon Gene Co., Ltd., Toyama, Japan) and the miRNeasy Mini Kit (Qiagen). For normalization of sample-to-sample variation in the RNA isolation step, 25 fmol of *Caenorhabditis elegans* cel-miR-39-3p (mirVana miRNA mimic; Thermo Fisher Scientific, Waltham, MA, USA) was added to each denatured sample mixed with lysis buffer. Reverse transcription was carried out using the TaqMan MicroRNA Reverse Transcription Kit (Thermo Fisher Scientific) and specific primers (hsa-miR-4442, Assay ID: 463327_mat; hsa-miR-3187-3p, Assay ID: 245619_mat; cel-miR-39-3p, Assay ID: 000200; Thermo Fisher Scientific), and qRT-PCR was performed using THUNDERBIRD Probe qPCR Mix (TOYOBO Co., Ltd., Osaka, Japan) in an ABI Prism 7900HT system (Thermo Fisher Scientific) according to the manufacturer’s instructions. Cycle threshold (Ct) values were calculated using the SDS 1.4 software (Thermo Fisher Scientific), and miRNA expression was normalized to cel-miR-39-3p using the ∆∆Ct method.

### Statistical analysis

Microarray data were analyzed using Microsoft Excel (Microsoft, Redmond, WA, USA). Welch’s *t* test was applied to compare the numbers of miRNAs, and paired *t* test was used to compare samples taken from patients before and after treatment. Statistical analysis of miRNA expression was performed with R (version 3.2.3; available from https://www.r-project.org/). The Wilcoxon signed-rank test was used to identify differentially expressed miRNAs in paired samples before and after treatment, and Steel’s test was used to compare the expression of miRNA in PM/DM patients with that in RA and SLE patients or healthy individuals. The Spearman rank correlation was used to analyze the correlation of the level of miRNA and the disease activities. *P* values of less than 0.05 were considered statistically significant.

## Results

### miRNA levels in plasma of PM/DM patients before and after treatment

**Fig. 1:**
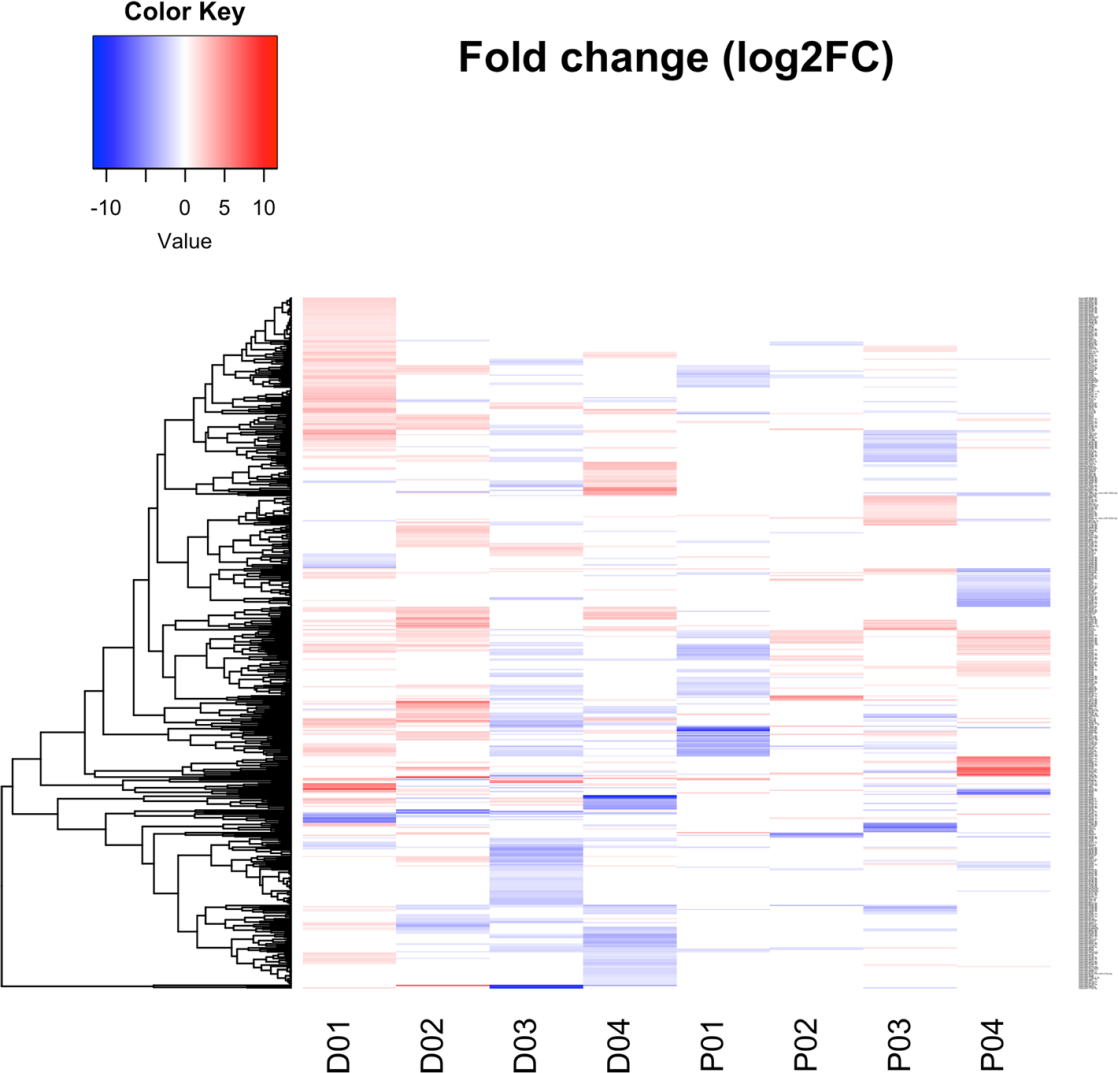
Plasma miRNA levels in patients with polymyositis/dermatomyositis before and after treatment. Plasma samples of patients with polymyositis (P) and dermatomyositis (D) were taken before and after treatment and analyzed by miRNA microarray; differentially expressed miRNAs were selected based on at least twofold change between paired samples taken before and after therapy, and a heat map was constructed using log_2_ fold change values (blue, the lowest, and red, the highest). miRNAs are indicated on the left; D01–D04: DM patients; P01–P04: PM patients

**Fig. 2:**
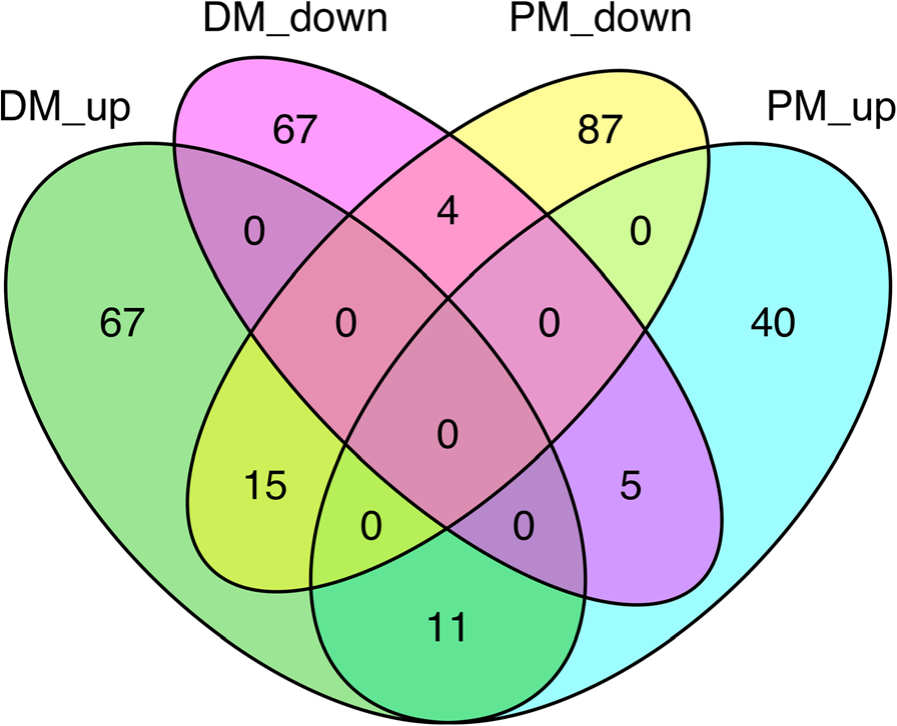
Numbers of upregulated and downregulated miRNAs in PM/DM after treatment. miRNA expression levels before and after treatment in DM and PM patients were averaged, and miRNAs were selected using the twofold filtering criterion. The Venn diagram shows the numbers of upregulated and downregulated miRNAs in PM and DM

Analysis of circulating extracellular miRNA in patients with myositis revealed differences in miRNA plasma levels between DM and PM patients and showed that treatment significantly affected miRNA expression in plasma (Fig. 1). The numbers of differentially expressed miRNAs with at least twofold change caused by treatment indicated that there were significantly more miRNAs whose plasma levels were affected by treatment in DM compared to PM (*P* = 0.0093) (Table 2). The average numbers of miRNAs differentially expressed in plasma of DM and PM patients before and after treatment are presented as the Venn diagram (Fig. 2), which showed the difference between DM and PM regarding the treatment effect. There were 11 upregulated and 4 downregulated miRNAs common for DM and PM (Table 3); among them, the expression of hsa-miR-3187-3p was significantly upregulated and that of hsa-miR-4442 was significantly downregulated (*P* = 0.023 and *P* = 0.016, respectively).

**Table 2:** Numbers of differentially expressed (twofold change) miRNAs in PM and DM patients

	D01	D02	D03	D04	P01	P02	P03	P04	*P* value
Upregulated (*n*)	213	128	27	75	11	37	63	57	0.18
Downregulated (*n*)	30	39	167	97	93	20	76	53	0.55
Total count (*n*)	243	167	194	172	104	57	139	110	0.0093*

*D01–D04* DM patients, *P01–P04* PM patients. *P < 0.05 was considered significant

**Table 3:** Changes of miRNA expression in PM/DM patients after treatment

miRNA	Fold change	*P* value
Upregulated		
hsa-miR-2278	2.43	0.27
hsa-miR-3175	13.60	0.26
hsa-miR-3187-3p	2.93	0.023 *
hsa-miR-331-3p	3.75	0.45
hsa-miR-3714	5.66	0.080
hsa-miR-4433b-3p	3.01	0.13
hsa-miR-451a	3.87	0.13
hsa-miR-498	2.67	0.15
hsa-miR-6073	5.69	0.061
hsa-miR-6790-3p	10.28	0.39
hsa-miR-6815-5p	2.35	0.21
Downregulated		
hsa-miR-28-5p	0.19	0.83
hsa-miR-4442	0.47	0.016*
hsa-miR-6826-5p	0.32	0.064
hsa-miR-7106-5p	0.16	0.21

miRNAs common for PM and DM are shown. *P < 0.05 was considered significant

### Validation of hsa-miR-3187-3p and hsa-miR-4442 expression in plasma

**Fig. 3:**
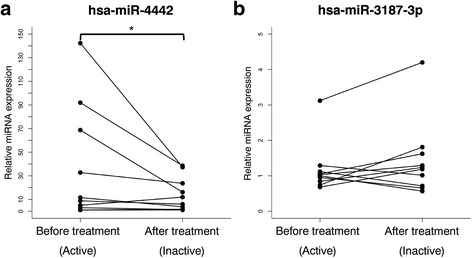
Validation of hsa-miR-4442 and hsa-miR-3187-3p expression in plasma of PM/DM patients. Quantitative RT-PCR was performed to confirm the differential expression of has-miR-4442 and hsa-miR-3187-3p in plasma of before (active) and after treatment (inactive) patients (*n* = 10). **a** The expression of hsa-miR-4442 was significantly decreased after treatment (*P < 0.05). **b** There was no significant difference in the expression of hsa-miR-3187-3p

To validate the differential expression of hsa-miR-3187-3p and hsa-miR-4442, we performed qRT-PCR which confirmed that the levels of plasma hsa-miR-4442 were markedly decreased by treatment (*P* = 0.047). However, there was no significant difference in the expression of hsa-miR-3187-3p (*P* = 0.28) (Fig. 3). These results indicate that hsa-miR-4442 may be associated with PM/DM.

### hsa-miR-4442 expression in plasma from patients with active PM/DM, RA, and SLE, and healthy individuals

**Fig. 4:**
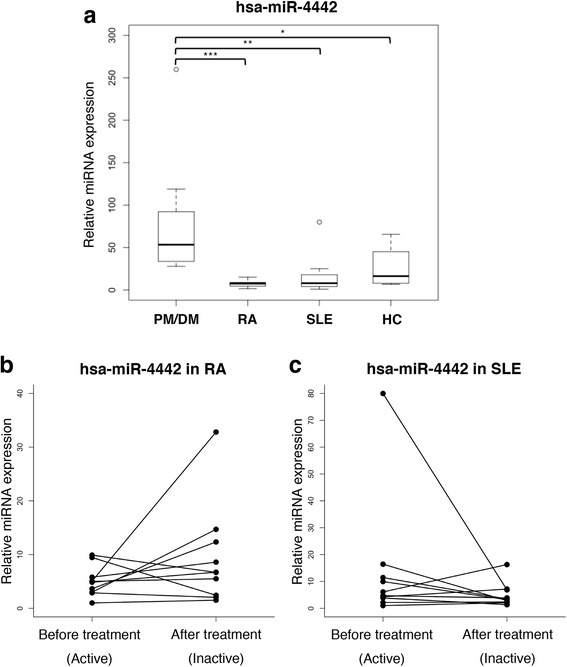
**a** Comparison of plasma hsa-miR-4442 expression between active PM/DM and RA, SLE, and healthy individuals using qRT-PCR. The expression of hsa-miR-4442 was significantly higher in PM/DM compared to RA, SLE, or healthy controls (*P < 0.05, **P < 0.01, and ***P < 0.001, respectively). **b**, **c** Quantitative RT-PCR was performed to confirm the differential expression of has-miR-4442 in plasma of before (active)/after treatment (inactive) RA and SLE patients. There was no statistically significant difference in either patient. PM/DM: polymyositis and dermatomyositis (*n* = 10); RA: rheumatoid arthritis (*n* = 10); SLE: systemic lupus erythematosus (*n* = 12); HC: healthy control (*n* = 10)

To verify the relationship between plasma hsa-miR-4442 levels and PM/DM, we compared the expression of this miRNA in PM/DM patients with that in patients with other autoimmune diseases (RA and SLE) and healthy individuals. Plasma expression of hsa-miR-4442 was significantly higher in active PM/DM compared with active RA (*P* = 0.00045), active SLE (*P* = 0.0011), or healthy control (*P* = 0.028) (Fig. 4a), further supporting the notion that circulating extracellular hsa-miR-4442 may be associated with PM/DM. We additionally analyzed the differential expression of hsa-miR-4442 in active/inactive SLE and RA patients using qRT-PCR (Fig. 4b, c). There was no statistically significant difference in either patient.

### Correlation of hsa-miR-4442 level in plasma and disease activities

We examined the correlation between the plasma hsa-miR-4442 level and disease activities. The level of miRNA was positively correlated with Skeletal Disease Activity in MITAX (Table 4). There was a weak positive correlation with ESR. There was no correlation with MITAX total score, CK, ALD, and LDH.

**Table 4:** Correlation of hsa-miR-4442 level in plasma and disease activities

	ρ	*P* value
MITAX		
Constitutional disease activity	0.081	0.73
Cutaneous disease activity	− 0.025	0.92
Skeletal Disease Activity	0.47	0.038*
Gastrointestinal disease activity	− 0.099	0.68
Pulmonary disease activity	0.046	0.85
Cardiovascular disease activity	− 0.058	0.81
Muscle disease activity	0.16	0.49
Total score	0.15	0.54
CK (IU/L)	− 0.017	0.95
ALD (IU/L)	0.083	0.73
LDH (IU/L)	− 0.068	0.78
ESR (mm/h)	0.37	0.10

^*^P < 0.05 was considered significant

## Discussion

Serum or plasma miRNAs have been reported to be associated with RA [18], SLE [19], and other autoimmune diseases [20]. The current study was the first to compare plasma miRNA expression in PM/DM patients before and after treatment using miRNA microarray. We found a difference in plasma miRNA profiles between PM and DM patients and identified several miRNAs whose plasma levels were affected by treatment (Figs. 1 and 2). A recent study revealed differential expression of serum miRNAs between PM and DM, showing significant association of three miRNAs (hsa-miR-3676, hsa-miR-3907, and hsa-miR-877*) with disease activity [21]. However, we did not observe changes in these miRNAs in our study, suggesting that miRNA spectra in serum and plasma of PM/DM patients may be different.

There were some reports about the miRNA level in skin and muscle in inflammatory myopathies, and we compare our plasma data with these reports. Inoue et al. showed that skin tissue miRNA array analysis demonstrated that the hsa-miR-223 level was markedly decreased in Gottron’s papules of DM and CADM [22]. The transfection of a specific inhibitor of hsa-miR-223 in keratinocytes led to upregulation of the PKCε protein and resulted in abnormally increased cell proliferation. The serum hsa-miR-223 concentration was decreased in PM/DM patients, particularly in CADM (clinically amyopathic dermatomyositis) patients, compared with healthy controls. The expression of plasma hsa-miR-223 in our study seemed to be increased after treatment. However, the increase was not significant. This result could support that low level of hsa-miR-223 was associated with skin lesion and increased in parallel with treatment. hsa-miR-1, hsa-miR-133a, hsa-miR-133b, and hsa-miR-206 are critical regulators of myoblast-to-myocyte differentiation through regulation of multiple genes [23]. Georgantas et al. observed decreased expression of hsa-miR-1, hsa-miR-133a, and hsa-miR-133b in PM/DM and inclusion body myositis (IBM), as well as decreased expression of miR-206 in DM [24]. TNFα was significantly inversely correlated with decreased myogenic miRNA expression in the inflammatory myopathy subtypes. These miRNAs could not be detected in our miRNA array analysis. These miRNAs in muscle tissue may not be released into the blood.

Recently, the classification of PM/DM was being changed by the concept of viewing inflammatory myopathies as a spectrum, with muscle and skin involvement occurring to varying degrees [25]. ADM (amyopathic dermatomyositis) is on the pure skin portion of the spectrum, while HDM (hypomyopathic dermatomyositis) is slightly closer to the muscle side. CDM (classic dermatomyositis) is in the middle of the spectrum and PM have purely muscle disease with a different pathologic process from ADM, HDM and CDM. Furthermore, the subsets of autoantibody status (such as anti-synthetase antibodies, Mi-2, SRP, and others) were increasingly being recognized as an improved way to phenotype patients with inflammatory myopathies [26]. It is possible that the difference of miRNA profiles between PM and DM is involved in the appearance of these spectrum, phenotypes, and autoantibody status. In this study, since the number of available samples was not sufficient, only miRNAs commonly fluctuated in PM/DM were analyzed. We thought that these miRNAs might be involved in common pathology to PM/DM.

Our results indicate that the expression of plasma hsa-miR-4442 was significantly decreased after treatment (Fig. 3) and that it was significantly higher in PM/DM compared to RA, SLE, or healthy controls (Fig. 4a). The change of hsa-miR-4442 could not be denied the possibility of merely the influence of the drug; however, the fluctuations of SLE and RA seemed to be not as much as PM/DM (Fig. 4b, c). Therefore, we believe that the change may not be caused just by the drug. There has been no report on the function of hsa-miR-4442 or its association with a disease in general. Furthermore, we confirmed a positive correlation between plasma has-miR-4442 level and Skeletal Disease Activity in MITAX and a weak positive correlation with ESR (Table 4). However, we could not find any correlation between hsa-miR-4442 expression level and serum autoantibody profiles (Additional file 1: Table S1). Thus, this is the first study to reveal possible correlation of the plasma hsa-miR-4442 level with a pathological condition (myositis), suggesting that it may be a candidate biomarker for PM/DM diagnosis or disease activity.

However, this study had limitations. First, the sample size was small, which might have caused a type 2 error in statistical analysis. Therefore, the role of hsa-miR-4442 in PM/DM suggested here should be confirmed in studies using larger patient cohorts. Also, we did not investigate the cellular source of plasma hsa-miR-4442 or its functional role in the pathogenesis of PM/DM, as well as the mechanism underlying the decrease of plasma hsa-miR-4442 content after treatment. These important aspects should be investigated to clarify the significance of hsa-miR-4442 in the development and progression of autoimmune myositis.

## Conclusions

In summary, we found differences in plasma miRNA profiles between PM and DM and between active and inactive PM/DM. Among the differentially expressed plasma miRNAs, has-miR-4442 levels were confirmed to be significantly lower after treatment, while being significantly higher in active PM/DM compared with RA, SLE, or healthy individuals. This is the first report about plasma miRNA profiling in PM/DM patients, laying a foundation for further studies to reveal the role of miRNAs in PM/DM.

## Additional file

Additional file 1: Serum autoantibody profiles of PM/DM patients in this study. (DOCX 51 kb)

## Abbreviations

ADM: Amyopathic dermatomyositis
CADM: Clinically amyopathic dermatomyositis
CDM: Classic dermatomyositis
HDM: Hypomyopathic dermatomyositis
miRNA: MicroRNA
MITAX: Myositis Intention to Treat Activity Index
PM/DM: Polymyositis and dermatomyositis
qRT-PCR: Quantitative reverse transcription polymerase chain reaction
RA: Rheumatoid arthritis
SLE: Systemic lupus erythematosus
