# Supplementary material for: Circulating plasma microRNA profiling in patients with polymyositis/dermatomyositis before and after treatment: miRNA may be associated with polymyositis/dermatomyositis
Source: Inflamm Regen. 2018 Jan 8;38:1. doi: 10.1186/s41232-017-0058-1 (PMC5757292; doi:10.1186/s41232-017-0058-1)
Supplement: Additional file 1: — Serum autoantibody profiles of PM/DM patients in this study. (DOCX 51 kb) [file 41232_2017_58_MOESM1_ESM.docx]

|  | Patients | Mi-2 | Ku | PM-Scl100 | PM-Scl75 | Jo-1 | SRP | PL-7 | PL-12 | EJ | OJ | Ro-52 |
| --- | --- | --- | --- | --- | --- | --- | --- | --- | --- | --- | --- | --- |
| PM | P01 | - | - | - | - | - | - | - | + | - | + | +/- |
|  | P02 | - | - | - | - | - | - | - | - | - | - | - |
|  | P03 | - | - | - | - | +/- | - | 2+ | - | - | - | 3+ |
|  | P04 | - | 3+ | - | +/- | - | + | - | +/- | - | - | +/- |
|  | P05 | - | - | - | - | - | - | - | - | - | - | - |
| DM | D01 | - | - | - | - | 3+ | - | - | - | - | - | 3+ |
|  | D02 | - | - | - | - | - | +/- | - | +/- | - | - | - |
|  | D03 | 2+ | - | - | - | - | + | - | - | - | - | - |
|  | D04 | - | - | - | +/- | - | + | - | +/- | 3+ | - | 3+ |
|  | D05 | +/- | - | - | - | - | - | +/- | +/- | - | - | - |

**Supplementary Table1** serum autoantibody profiles in PM/DM patients

PM: polymyositis; DM: dermatomyositis
